# Cardiac tamponade associated with delayed ascending aortic perforation after blunt chest trauma: a case report

**DOI:** 10.1186/s12893-017-0266-2

**Published:** 2017-06-17

**Authors:** Dae Woong Ryu, Mi Kyung Lee

**Affiliations:** Department of Thoracic and Cardiovascular Surgery, School of Medicine, Wonkwang University, 460 Iksandae-ro, Iksan, Jeonbuk 570-749 Republic of Korea

**Keywords:** Cardiac tamponade, Delayed aortic injury, Rib fracture, Blunt chest trauma

## Abstract

**Background:**

Cardiac tamponade due to aortic injury after blunt trauma is a rare and potentially fatal injury. Most aortic injuries caused by blunt trauma present as aortic dissection or rupture of the aortic isthmus. Several cases of delayed aortic injury have been reported. However, all of these injuries were observed in the descending aorta because they had been caused by a posterior rib fracture.

**Case presentation:**

We report the first case of cardiac tamponade associated with delayed ascending aortic perforation 2 weeks after blunt trauma. The patient was an 81-year-old man.

**Conclusion:**

In cases of blunt chest trauma, delayed ascending aortic injury causing cardiac tamponade is possible associated with various causes such as direct injury by fractured rib or delayed aortic perforation of initial blunt injury.

## Background

**Fig. 1:**
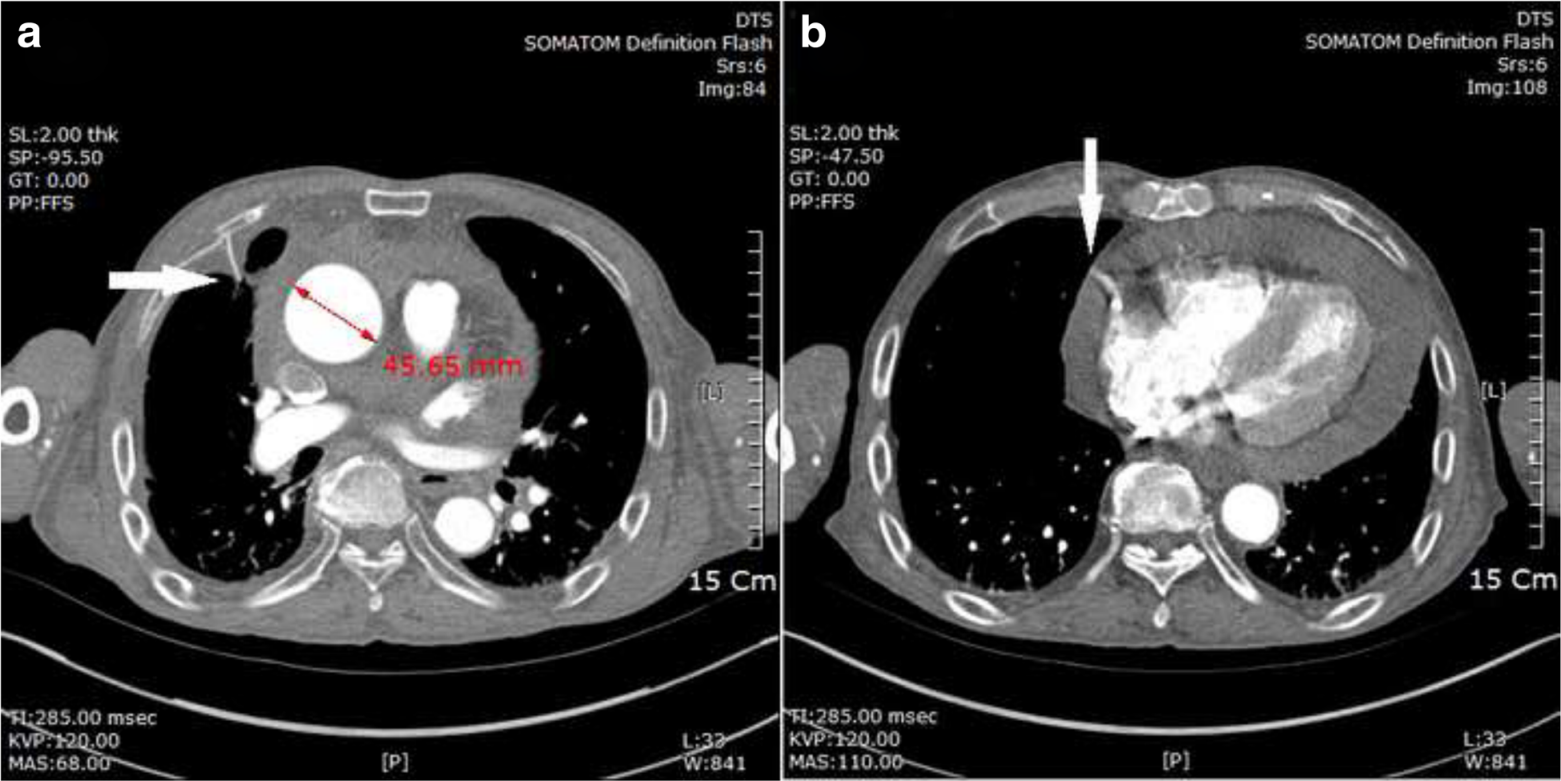
**a** Bony fragment of the fractured 4th rib (*arrow*). The ascending aorta was measured to be about 45 mm in maximum diameter. **b** Contrast-enhanced computed tomography showing collection of hemorrhagic fluid in the pericardium, with enhancement in the *right side* of the heart, suggesting leakage of contrast medium (*arrow*)

**Fig. 2:**
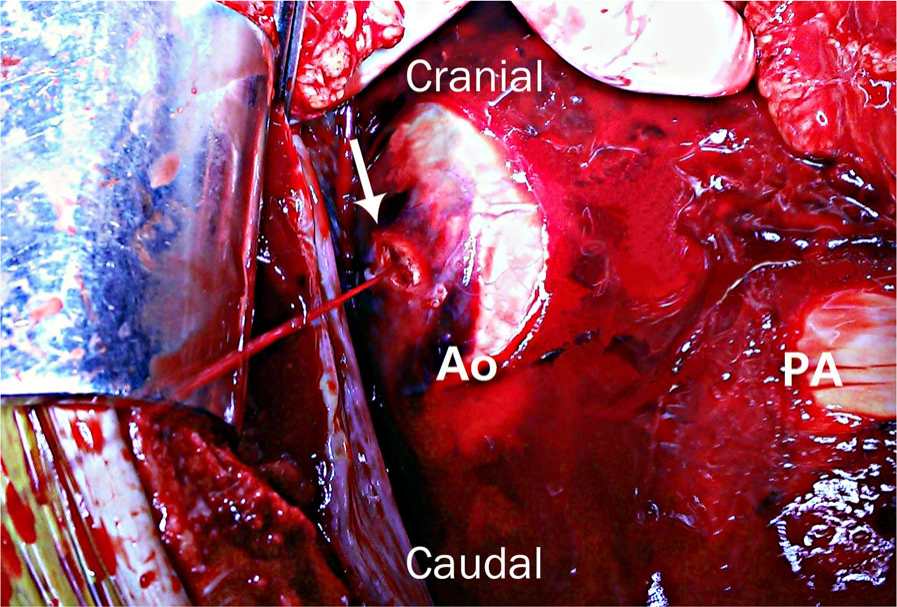
A laceration-type wound approximately 7 mm in size with 2 mm perforation on the right side of the ascending aorta was found at the level of perforated pericardium (*arrow*)

Cardiac tamponade after blunt trauma is rare and usually associated with rupture of cardiac chambers on the relatively weaker right side [1]. Aortic injury causing tamponade after blunt trauma is an even rarer catastrophic injury that leads to death. Blunt trauma can inflict injury to the aorta through several mechanisms. Most aortic injuries caused by blunt trauma present as aortic dissection or rupture of the aortic isthmus due to indirect forces. Direct injury of the aorta can sometimes be caused by a fractured bone fragment [2]. Several cases of delayed aortic injury caused by rib fracture have been reported. However, almost all cases involved the descending aorta owing to a posterior rib fracture. In our case, a laceration-type wound approximately 7 mm in size with 2 mm perforation (Fig. 2) was found at the level of perforated pericardium (Fig. 1a). To our knowledge, delayed ascending aortic perforation after blunt trauma has not been reported. We describe a case of cardiac tamponade associated with delayed ascending aortic perforation 2 weeks after blunt trauma.

## Case presentation

An 81-year-old man presented to our hospital with chest pain and dyspnea 2 weeks after sustaining blunt trauma from a cultivator accident. At the time of the accident, he did not seek medical care. On admission, he was alert and complained of chest pain. He had a history of hypertension and hyperlipidemia which was being treated with medications for 9 years. A bruise was noted on the anterior chest. His blood pressure had decreased to 70/50 mmHg; however, his other vital signs were normal.

Laboratory tests revealed decreased hemoglobin (8.5 g/dL), elevated plasma lactate (104 mg/dL), and normal creatine kinase (CK), CK-MB, and troponin T levels. The other laboratory values were within the reference limits.

An electrocardiogram indicated sinus rhythm with premature atrial complexes and low voltage QRS complexes. Initial contrast-enhanced computed tomography (CT) showed a collection of hemorrhagic fluid in the pericardium and suspected leakage of contrast medium on the right ventricle (Fig. 1b), with multiple fractured ribs(3rd to 5th ribs) on the right aspect of the chest. The ascending aorta was measured to be about 45 mm in maximum diameter (Fig. 1a).

We suspected right-sided cardiac rupture, established an anesthetic plan based on the cardiac rupture and performed emergency surgery. After median sternotomy, immediately after pericardial incision, approximately 200 mL stagnated blood gushed out and the blood pressure normalized. After removing the hematoma covering the heart, continuous pulsatile flow of fresh blood was observed coming from the ascending aorta. A laceration-type wound approximately 7 mm in size with 2 mm perforation (Fig. 2) was closed with a 4–0 pledgeted polypropylene suture. We could not find any obvious laceration of the parietal pericardium except for a small perforation and an abrasion at the level of the ascending aortic injury. The tip of sharp bony fragment of the fractured 4th rib was removed using a rongeur. The patient was discharged after 14 days, without any complications.

## Discussion

Cardiac tamponade after blunt trauma is rare and associated with very high mortality. The main cause of tamponade is rupture of the cardiac chambers, predominantly on the relatively weaker right side of the heart [3]. However, cardiac tamponade caused by aortic injury after blunt trauma is extremely rare [1]. In our case, CT showed a collection of hemorrhagic fluid in the pericardium, suspected leakage of contrast medium from the right heart, and fractured ribs sufficiently distant from the heart (Fig. 1). Therefore, we initially assumed that delayed cardiac tamponade was caused by rupture in the right side of the heart.

Aortic injury after blunt trauma usually occurs in the extrapericardial part of the aorta, and only 15% of patients with an aortic injury after blunt trauma survive until hospital arrival. The mechanism of injury remains the most important factor, e.g., falls from >10 ft., motor vehicle accidents at speeds >30 mph, unrestrained drivers, ejected passengers, and pedestrians struck by motor vehicles [4].

Blunt trauma can inflict injury to the aorta through several mechanisms. The aorta can be damaged by indirect forces such as stretching, shearing due to deceleration loads, or increases in internal blood pressure. In such cases, the aortic isthmus is the most common site of rupture. A displaced, fractured thoracic vertebra, or bony intrusion by a fractured rib and clavicle, can cause direct injury to the aorta [2]

Multiple rib fractures are common in blunt chest trauma, and can lead to various injuries of intrathoracic organs such as pneumothorax, hemothorax, lung contusions, and injuries to neighboring organs [5]. A great vessel injury caused by a rib fracture can be fatal but, fortunately, seldom occurs [6].

However, several cases of delayed aortic injury caused by a rib fracture have been reported, and almost all patients had involvement of the descending aorta owing to a posterior rib fracture [5]. Because the ascending aorta is protected by the sternum and the ribs are relatively distant from the heart, as in our case, ascending aortic injury after blunt trauma is rarely reported in the literature. The cause of such delayed perforation is uncertain; however, we suggest that superficial injury of the aorta associated with a bony fragment or direct blunt injury occurred at the time of the accident but perforation was delayed. Continuous exposure to high pressure in the aorta may have led to stretching of the wound, and perforation eventually occurred 2 weeks later [7]. Alternatively, the physical movements of the patient might have caused a new laceration from a bony fragment [8].

If delayed aortic perforation had initiated from the intimal tear, arterial blood pressure would have forced blood between the layers of the aortic wall forming a false aneurysm [2]. However, in our case, false aneurysm was not found in CT scan. This suggests that the injury began from outside the aorta associated with a fractured rib or direct blunt injury.

Because the patient did not seek medical care at the time of the accident, we were not able to determine the exact cause. However, if physical movements caused a new laceration after the accident, we believe that removal of a bony fragment by means of video-assisted thoracoscopic surgery (VATS) could have prevented the perforation, as reported by Funaki et al. [9].

## Conclusion

Even in cases of blunt chest trauma without a fatal injury, delayed ascending aortic injury causing cardiac tamponade is possible associated with various causes such as direct injury by fractured rib or delayed aortic perforation of initial blunt injury. Therefore, careful follow-up is needed, and if periaortic bony fragments pose risks to the aorta, active preventive management such as rib resection by using VATS or thoracotomy may be able to prevent fatal outcomes.

## Abbreviations

CK: Creatine kinase
CT: Computed tomography
VATS: Video-assisted thoracoscopic surgery
